# Scan-rescan reproducibility of quantitative assessment of inflammatory carotid atherosclerotic plaque using dynamic contrast-enhanced 3T CMR in a multi-center study

**DOI:** 10.1186/s12968-014-0051-7

**Published:** 2014-08-01

**Authors:** Huijun Chen, Jie Sun, William S Kerwin, Niranjan Balu, Moni B Neradilek, Daniel S Hippe, Daniel Isquith, Yunjing Xue, Kiyofumi Yamada, Suzanne Peck, Chun Yuan, Kevin D O’Brien, Xue-Qiao Zhao

**Affiliations:** 1 Department of Biomedical Engineering, Center for Biomedical Imaging Research, School of Medicine, Tsinghua University, Beijing 100084, China; 2 Department of Radiology, University of Washington, 850 Republican St, Seattle 98109, WA, USA; 3 The Mountain-Whisper-Light Statistics, 1827 23rd Ave. East, Seattle 98112, WA, USA; 4 Division of Cardiology, University of Washington School of Medicine, 325 9th Ave, Harborview Medical Center, Seattle 98104, WA, USA

**Keywords:** Carotid artery, Atherosclerosis, Reproducibility, Dynamic contrast-enhanced cardiovascular magnetic resonance

## Abstract

**Background:**

The aim of this study is to investigate the inter-scan reproducibility of kinetic parameters in atherosclerotic plaque using dynamic contrast-enhanced (DCE) cardiovascular magnetic resonance (CMR) in a multi-center setting at 3T.

**Methods:**

Carotid arteries of 51 subjects from 15 sites were scanned twice within two weeks on 3T scanners using a previously described DCE-CMR protocol. Imaging data with protocol compliance and sufficient image quality were analyzed to generate kinetic parameters of vessel wall, expressed as transfer constant (*K*^*trans*^) and plasma volume (*v*_*p*_). The inter-scan reproducibility was evaluated using intra-class correlation coefficient (ICC) and coefficient of variation (CV). Power analysis was carried out to provide sample size estimations for future prospective study.

**Results:**

Ten (19.6%) subjects were found to suffer from protocol violation, and another 6 (11.8%) had poor image quality (n = 6) in at least one scan. In the 35 (68.6%) subjects with complete data, the ICCs of *K*^*trans*^ and *v*_*p*_ were 0.65 and 0.28, respectively. The CVs were 25% and 62%, respectively. The ICC and CV for *v*_*p*_ improved to 0.73 and 28% in larger lesions with analyzed area larger than 25 mm^2^. Power analysis based on the measured CV showed that 50 subjects per arm are sufficient to detect a 20% difference in change of *K*^*trans*^ over time between treatment arms with 80% power without consideration of the dropout rate.

**Conclusion:**

The result of this study indicates that quantitative measurement from DCE-CMR is feasible to detect changes with a relatively modest sample size in a prospective multi-center study despite the limitations. The relative high dropout rate suggested the critical needs for intensive operator training, optimized imaging protocol, and strict quality control in future studies.

## Background

Neovasculature distributed inside the atherosclerotic plaque is the primary route of inflammatory cell infiltration, a key process for plaque initiation, progression, and rupture [1]. Plaque neovascularization and inflammation are associated with clinical cardiovascular events caused by atherosclerosis [2]–[4]. As a result, inflammation is an emerging target for treatment in atherosclerosis [5],[6]. To assess and monitor the therapeutic effects of new treatments, imaging techniques are needed that detect and quantify neovascularization and inflammation in-vivo.

Dynamic Contrast-Enhanced (DCE) cardiovascular magnetic resonance (CMR) can quantify atherosclerotic angiogenesis and inflammation in-vivo by measuring the density and function of the neovasculature through kinetic modeling. Kinetic parameters, including fractional plasma volume (*v*_*p*_), and the transfer constant (*K*^*trans*^), have been found to be associated with histological measurements of plaque neovasculature and macrophages [7]–[9]. Based on this knowledge, DCE-CMR has been successfully used in assessment of the therapeutic response of the vasa vasorum in patients with atherosclerotic plaque, including a decrease in *K*^*trans*^ after 1-year intensive lipid therapy [10]. Another study demonstrated the effect of pioglitazone on atheroma by using DCE-CMR in an animal model [11]. DCE-CMR was also utilized to monitor the nature progression of atherosclerotic plaque [12].

For such studies, reproducibility of quantitative parameters is key to study planning. However, the reproducibility of the *K*^*trans*^ and *v*_*p*_ measurements of human vessel wall remains largely unknown, especially for clinical studies involving multiple imaging centers. This study sought to investigate the inter-scan reproducibility of kinetic parameters in atherosclerotic plaque using dynamic contrast-enhanced (DCE) CMR in a multi-center setting at 3T.

## Methods

### Study population

This study was performed with institutional review board approval at each participating clinical site. Written informed consent was obtained prior to study procedures. Subjects who were enrolled in the Atherothrombosis Intervention in Metabolic syndrome with low HDL/high triglycerides: Impact on Global Health outcomes (AIM-HIGH) trial [13] were asked for willingness to participate in a MR reproducibility study. They were 45 and older, with dyslipidemia and clinically established atherosclerotic disease in coronary, cerebrovascular/carotid and/or peripheral arteries [13]. Subjects with any contraindication for MR examination or contrast injection were not included. Consented subjects underwent two carotid MR examinations with two weeks, which included a previously described DCE-CMR protocol. The present study, as well as most previous studies, used a bright-blood sequence for DCE-CMR [7]–[10], which does not allow assessment of near-normal arteries due to concerns of luminal signal contamination. Therefore, only subjects with distinct carotid plaques were included, defined as maximum wall thickness > 1 mm [14] measured on pre-contrast T1-weighted MR with a customized software (CASCADE) [15]. To ensure a multicenter design, each imaging site was restricted to a maximum of 6 subjects. From March 2009 to May 2012, of 47 subjects screened, 33 AIM-HIGH subjects were enrolled at 10 imaging sites. We also screened 21 subjects and enrolled 18 subjects with the same inclusion and exclusion criteria from 5 non-AIM-HIGH imaging sites to further increase the number of sites and subjects. In total, 51 subjects from 15 imaging sites were included.

This study was approved by the local IRB/RECs at each participating clinical site. A signed informed consent was obtained from participants.

### Magnetic resonance imaging

All subjects were scanned twice within two weeks on 3T MR scanners (GE HealthCare or Philips Healthcare) to acquire carotid artery images using commercially available carotid phased-array coils (GE: 6-channel, Neocoil LCC, Pewaukee, WI, USA; Philips: 8-channel, SHCG, Shanghai, China). Each reproducibility scan was conducted on the same scanner platform as the baseline scan. The imaging protocol included an axial multi-slice 2D spoiled gradient recalled echo sequence to acquire DCE images. On GE scanners 8 contiguous slices were acquired, centered on the bifurcation of the index carotid artery to ensure the alignment between repeated scans. The acquisition parameters were: field of view: 160*160 mm, matrix 256*256, imaging resolution: 0.625*0.625 mm, reconstructed image size 512*512, 2 mm slice thickness, repetition time 117 ms, echo time 5 ms, flip angle 50°. On Philips scanners, the protocol was slightly different to ensure a similar coverage, signal-to-noise ratio (SNR) and temporal/spatial resolution: only 4 contiguous slices were acquired, also centered on the bifurcation of index side, with 3 mm slice thickness, repetition time 126 ms. The index side was defined as the side with larger plaque. Coincident with the third dynamic scan in the sequence, 0.05 mmol/kg of a gadolinium-based contrast agent (Magnevist, Bayer Healthcare) was injected at a rate of 0.7 ml/s by a power injector. The contrast dose and injection rate was selected to avoid possible flow artifacts and weaken the requirement of high temporal resolution in DCE imaging [9]. Images were acquired at 18 time points separated by a repetition interval of 18 s. To impose a T1-dependent signal on inflowing blood, a spatial saturation band was used.

### Image analysis

**Figure 1:**
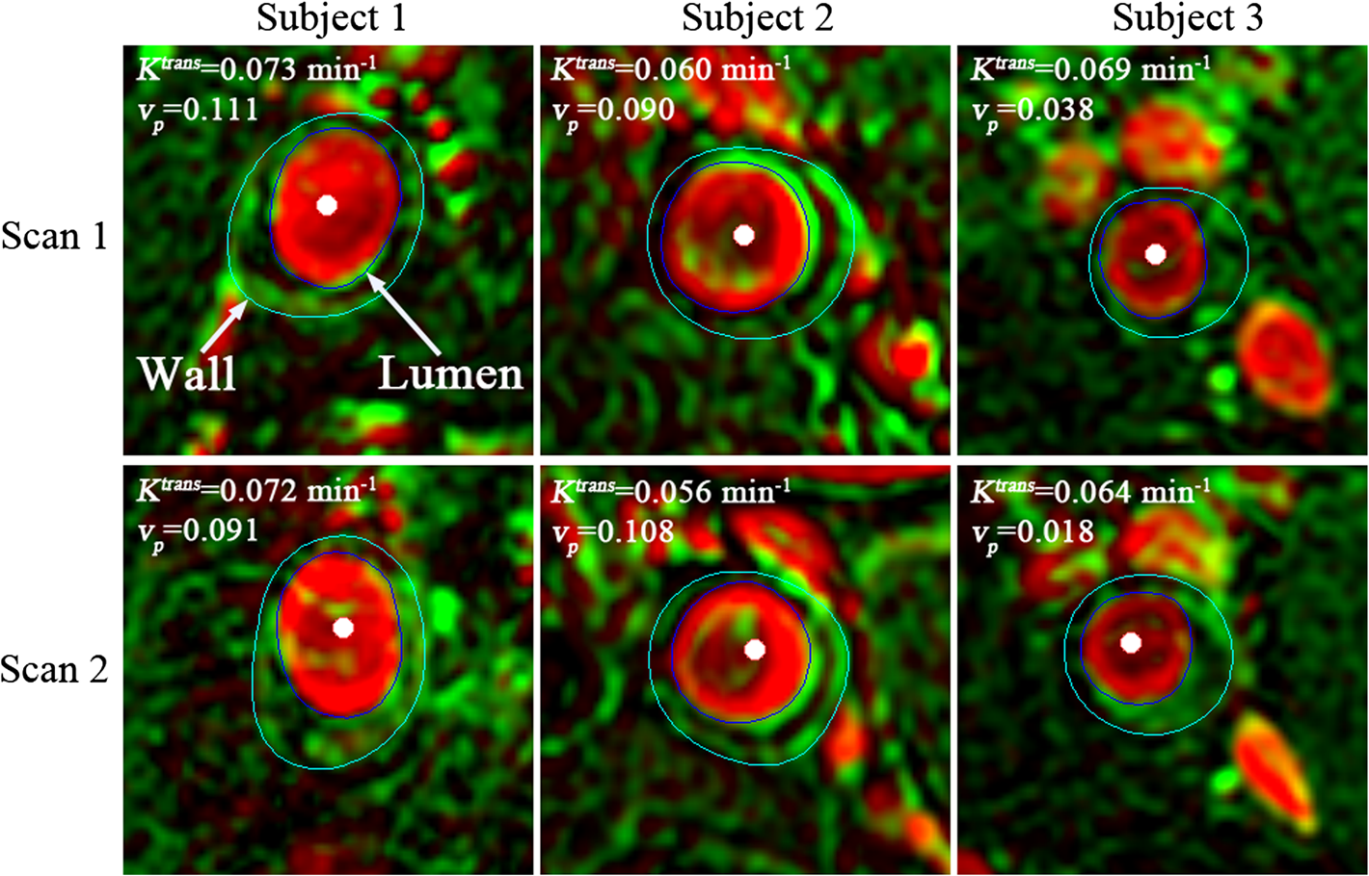
**Representative scan-rescan V-V images (rows) of three plaques (columns).** The white points indicate the carotid lumen; the white arrows point to the outlined lumen and outer wall boundaries. Green indicates *K*^*trans*^ with a range from 0 to 0.2 min^−1^ and red indicates *v*_*p*_ with a range from 0 to 1. Minor mis-resgistrations were occasionally present. The *K*^*tran*^ and *v*_*p*_ values shown in each figure are the measurements of the whole plaque.

A custom software (CASCADE) [15] was used for image analysis. The DCE-CMR images were analyzed to generate vasa vasorum (V-V) images as previously described [16]. This included registration and smoothing using the Kalman filtering registration and smoothing algorithm, extraction of the arterial input function, and calculation of *K*^*trans*^ and *v*_*p*_ for each pixel based on the Patlak kinetic model [17]. The resulting color-coded, parametric V-V images show *K*^*trans*^ in green and *v*_*p*_ in red (Figure 1), allowing single channel (green or red) to be displayed. Next, the V-V image was outlined to define the lumen boundary (the border of high *v*_*p*_ values within the lumen) and the outer wall boundary (the rim of high *K*^*trans*^ values representing adventitial enhancement) [18] (Figure 1).

Each scan was analyzed independently, blinded to the other scan and subject information. First, one trained reviewer (J. S., 3 years experiences in plaque analysis) identified all the boundaries on V-V images after checking the protocol compliance. Then, a second reviewer (H. C., 4 years experiences in plaque analysis) performed peer-review of the boundaries defined by the primary reviewer. Cases in which there was disagreement were discussed with the primary reviewer to reach the consensus opinion.

Lastly, *K*^*trans*^ and *v*_*p*_ measurements were calculated by averaging all pixels within the vessel wall, except for those within 1 mm of the lumen contour (excluded automatically by CASCADE). This exclusion minimized any influence from the high intensity lumen signal in bright-blood DCE images due to partial volume effects, blurring, and motion.

### Statistical analysis

The means of the scan 1 and scan 2 *K*^*trans*^ and *v*_*p*_ values were compared using the paired t-test. The overall between-scan reproducibility was estimated using a linear mixed model. The reproducibility results are presented as the between-scan standard deviation (SD), the between-scan CV (between-scan SD divided by the grand mean) [19] and the intra-class correlation (ICC). The 95% confidence intervals for these three reproducibility statistics were calculated using the non-parametric bootstrap. The differences in means and between-scan SDs across the GE and Phillips platforms were tested using the linear mixed model and the permutation test, respectively.

To explore the association of each subject’s between-scan reproducibility with their plaque area we plotted the subjects’ means and plaque sizes (the smaller of the two scans’ plaque areas) against the subjects’ between-scan SD. The association between the two factors and the between-scan SD was tested using the Spearman correlation (ρ) test. The least-squares fit was used to highlight the trends in the scatter plots.

For each kinetic measurement, the CV estimate was used to calculate sample sizes needed to detect differences in% changes (follow-up minus baseline, normalized by the baseline mean) between two treatment arms. The sample size calculations used the two-sided unpaired t-test with 80% power and the 0.05 significance level [20] without considering the exclusion rate. The CV for the change (follow-up minus baseline) was calculated from the CV for a single time measurement (obtained in the reproducibility analysis) by multiplying it by √2. This calculation assumes that the measurement error is the primary source of variability within each treatment group (most importantly that the expected treatment effect does not differ across patients).

The calculations were carried out in R (The R Foundation for Statistical Computing, Vienna, Austria), version 2.15.2. A p-value less than 0.05 was used to denote statistical significance.

## Results

Of the 51 subjects included in this study, 10 (19.6%) were excluded due to any significant violation of the DCE imaging protocol in at least one scan, including incorrect time interval (n = 3), too few time frames (n = 2), failure to inject contrast agent (n = 2) and improper alignment of images (n = 3). Additionally, 6 (11.8%) subjects were excluded for uninterpretable image quality in at least one scan, generally due to severe subject motion and/or low SNR. The remaining 35 (68.6%) subjects were available for reproducibility assessment. These subjects were from 14 sites with 2.5 ± 1.5 (mean ± standard deviation) per site range (1 to 6). Demographic and clinical characteristics of these subjects are given in Table 1.

**Table 1:** Demographic and clinical characteristics in 35 subjects included in reproducibility study

	**mean ± SD (range) or N (%)**
Age (years)	61 ± 8 (45–79)
Gender (female)	12 (34%)
History of Myocardial Infarction	9 (26%)
History of Stroke	3 (9%)
History of Hypertension	28 (80%)
History of Diabetes	10 (29%)
LDL-C (mg/dl)	81 ± 34 (33–205)
Triglycerides (mg/dl)	163 ± 57 (80–349)
HDL-C, (mg/dl)	35.3 ± 6.1 (20.0-49.8)

DCE-CMR protocol non-compliance and image quality issues were widely distributed across imaging sites. The 10 instances of protocol non-compliance were spread among 8 imaging sites and the 6 instances of poor image quality were spread among 5 sites. No site accounted for more than 2 exclusions due to protocol non-compliance and image quality combined. Only in one site, all the scanned subjects (2 subjects) were not included in the analysis due to protocol non-compliance.

**Figure 2:**
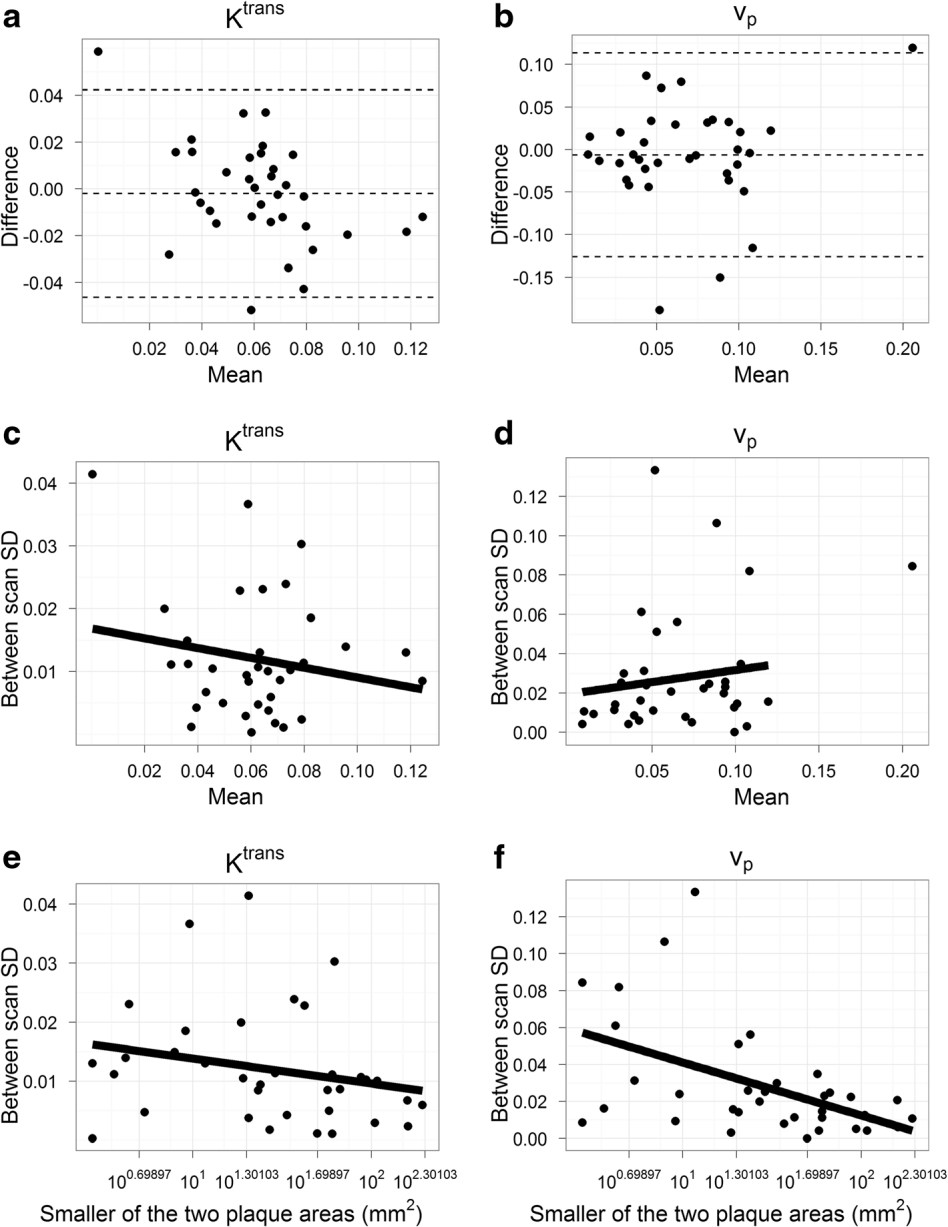
**Relationship of reproducibility with the mean measurement and with the plaque area. a**. Bland-Altman plot of *K*^*trans*^. **b**. Bland-Altman plot of *v*_*p*_. **c**. Per-subject mean *K*^*trans*^ (min^−1^) vs. per-subject between-scan SD of *K*^*trans*^ (ρ = −0.02, p = 0.9), **d**. Per-subject mean *v*_*p*_ (unitless) vs. per-subject between-scan SD of *v*_*p*_ (ρ = 0.25, p = 0.15). **e**. Plaque areas (on the log-scale) vs. per-subject between-scan SD of *K*^*trans*^ (ρ = −0.33, p = 0.06), **f**. Plaque areas (on the log-scale) vs. per-subject between-scan SD of *v*_*p*_ (ρ = −0.45, p = 0.007). Dashed lines in panel **(a)** and **(b)** indicate the mean differences and limits of agreement (±2 × SD of differences) for Bland-Altman plots. Solid lines in panels **(d-f)** are least square fits. Fitted line in panel **(d)** excludes one outlier with mean *v*_*p*_ above 0.20.

Figure 1 shows representative examples of the matched V-V images for two scans of three carotid arteries, showing *K*^*trans*^ in green and *v*_*p*_ in red. Mean *K*^*trans*^ and *v*_*p*_ for the first and second scan are compared in Table 2. Figure 2a and b show the Bland-Altman plots of *K*^*trans*^ and *v*_*p*_, respectively.

**Table 2:** Comparison of kinetic parameters across scans

	**Mean (Standard deviation)**		
	**Scan 1**	**Scan 2**	**p**^**‡**^
*K*^trans*^	0.061 (0.022)	0.063 (0.030)	0.6
*v*_*p*_^†^	0.064 (0.053)	0.070 (0.046)	0.5

^*^K^*trans*^: transfer constant in min^−1^.

^†^*v*_*p*_: plasma volume fraction (unitless).

^‡^paired t-test.

Overall reproducibility parameters for *K*^*trans*^, and *v*_*p*_ are shown in Table 3. For *K*^*trans*^, the CV was 25% (95% CI 15%-28%) and the ICC was 0.65 (95% CI 0.57-0.85) suggesting moderate reproducibility of that parameter. The parameter *v*_*p*_, on the other hand, exhibited substantially worse reproducibility with a CV of 62% (95% CI 36%-73%) and ICC of 0.28 (95% CI 0.14-0.70). The within-subject SD was not significantly related to the mean value for both *K*^*trans*^ (ρ = −0.02, p = 0.9, Figure 2c) and *v*_*p*_ (ρ = 0.25, p = 0.15, Figure 2d).

**Table 3:** Grand means, between-scan standard deviations (SDs) and coefficients of variation for kinetic parameters by vendor

	**Mean**	**SD**	**CV (%) (95% CI**^**‡**^**)**
*K*^trans*^			
All	0.062	0.015	25% (15%-28%)
GE	0.064	0.017	27% (16%-40%)
Philips	0.060	0.013	21% (10-22%)
*p*^§^	0.6	0.12	0.12
*v*_*p*_^†^			
All	0.067	0.042	62% (36%-73%)
GE	0.068	0.042	62% (46-73%)
Philips	0.066	0.040	60% (18-71%)
*p*^§^	0.8	0.9	0.9

^*^K^*trans*^: transfer constant in min^−1^.

^†^*v*_*p*_: plasma volume fraction (unitless).

^‡^CI : confidence interval.

^§^the linear mixed model test for the comparison of the means and the permutation test for the comparison of the SDs and CVs (NS : p > 0.05).

The poor performance of *v*_*p*_ had a significant association with plaque size exhibiting a negative Spearman correlation (ρ = −0.45, p = 0.007; Figure 2f). If subjects with analyzed areas smaller than 25 mm^2^ were excluded, leaving 19 subjects for analysis, the CV and ICC for *v*_*p*_ improved to 28% (95% CI 18%-31%) and 0.73 (0.67-0.89), respectively. The association between *K*^*trans*^ reproducibility and plaque size showed only marginal significance (ρ = −0.33, p = 0.06; Figure 2e). Little change in the ICC and CV of *K*^*trans*^ was observed for the 25 mm^2^ cutoff.

**Figure 3:**
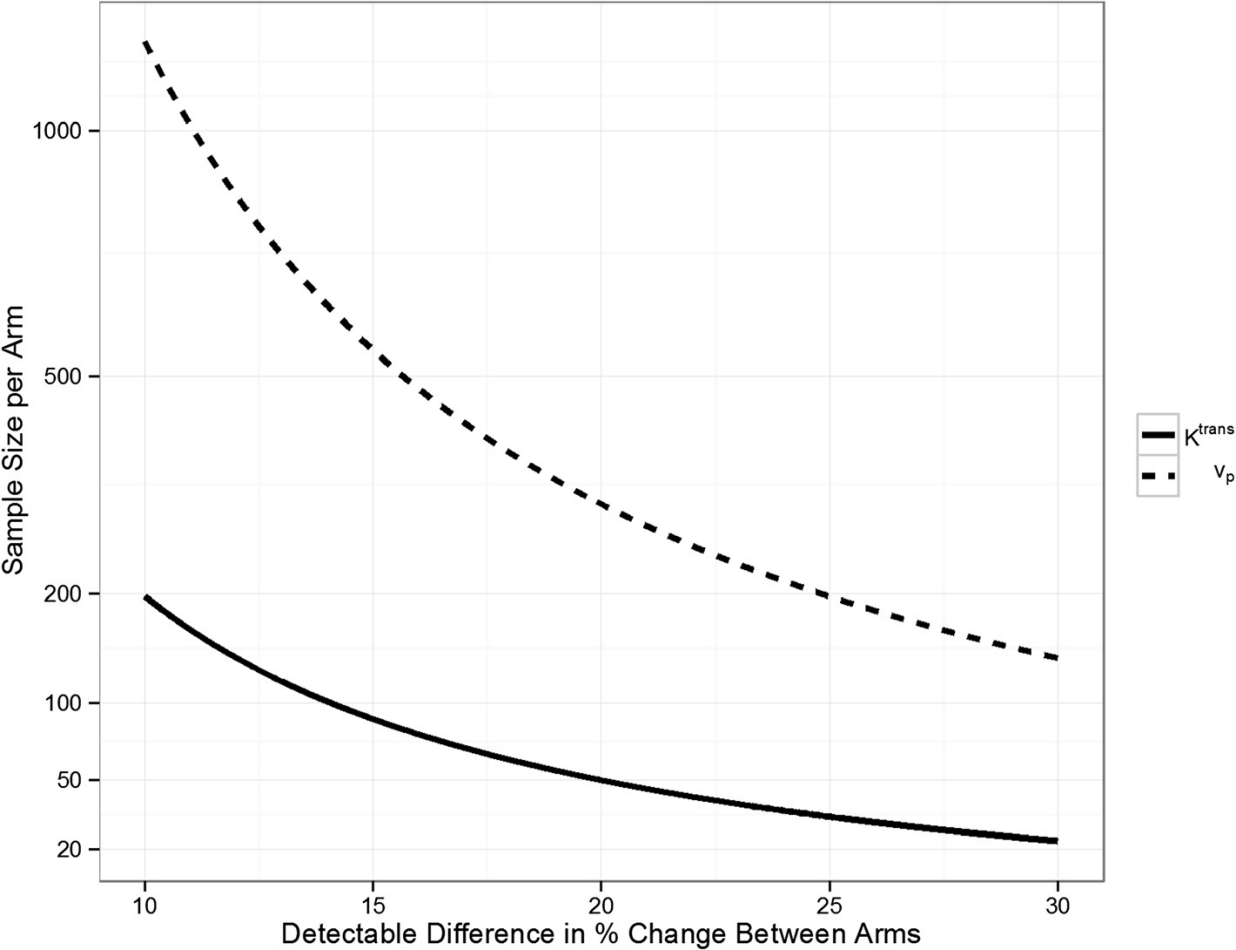
**Sample size analysis.** Sample size needed for each kinetic parameter to detect a difference in% change between treatment arms with 80% power using a two-sided unpaired t-test at the 0.05 significance level. The solid and dotted lines are the required sample sizes to detect changes from 10% to 30% for *K*^*trans*^ and *v*_*p*_, respectively.

Based on the reproducibility results above, the sample sizes needed to detect differences in change of each kinetic parameter between two treatment arms were computed without considering the exclusion rate (Figure 3). For K^*trans*^, 197, 50 and 23 subjects per arm would be needed to detect differences of 10%, 20% and 30%, respectively. For *ν*_*p*_, 1224, 307 and 137 subjects per arm would be needed, respectively. The required sample sizes for *ν*_*p*_ reduced to 254, 64, and 29 if subjects with analyzed plaque areas less than 25 mm^2^ were excluded.

The use of 2 primary scanner platforms (GE and Philips) allowed us to also evaluate whether these findings depended on vendor. In total, 20 subjects scanned on GE scanners were included in the study and 15 subjects scanned on Philips scanners were included. As summarized in Table 3, the differences in means, standard deviations and CVs of kinetic parameters across platforms were relatively small and not statistically significant.

## Discussion

This study provides an evaluation of scan-rescan reproducibility of kinetic parameters (*K*^*trans*^ and *v*_*p*_) generated from DCE-CMR using 3T MR scanners for multi-center studies of carotid atherosclerosis. We found that *K*^*trans*^ had moderate but acceptable reproducibility in the multi-center setting, with an overall CV of 25%. For this level of reproducibility, a 20% greater relative reduction in *K*^*trans*^ could be detected with 80% power and p = 0.05 using 50 subjects per arm. Notably, a single arm study involving 28 subjects on lipid lowering therapy using similar techniques reported slightly greater than 20% reduction in *K*^*trans*^ over a period of 1 year [10]. As a comparison for the reproducibility of vessel wall inflammation quantification, a widely used serum-based inflammation biomarker, high-sensitive C-Reactive Protein, showed a large test-retest variation with CV of 46.2% [21].

For *v*_*p*_, on the other hand, the reproducibility was lower, with an overall CV of 62%, indicating it is not a preferable parameter for clinical studies. The reason may be that *v*_*p*_, which represents tissue blood supply, is more vulnerable to signal influences from lumen. The much higher reproducibility, as shown in a subset of patients with larger plaques, supports this hypothesis. Notably, previous histological validation studies [7]–[9] were all on patients with large lesions, further indicating *v*_*p*_ is more reliable parameter in large plaques. Additionally, the limited temporal resolution may also introduce some variance for *v*_*p*_[22]. As such, future studies involving limiting *v*_*p*_ are suggested to use subjects with larger lesions.

Of note, the reproducibility of DCE-CMR parameters found in this multi-center experience is similar to that reported in a recent single-center study [9]. In that study, reported ICCs for *K*^*trans*^ and *v*_*p*_ were 0.79 and 0.48, respectively. CVs were 16% and 26%. The somewhat better reproducibility in that study may reflect the use of a single imaging center. In addition, their population included only subjects with at least 30% carotid stenosis, which may have enriched their study with larger, more reproducible lesions.

Another key finding of this study involved the use of both GE and Philips scanner platforms. Although each subject was scanned on the same scanner, the measurements and variability could have been platform dependent, because the two platforms required slightly different imaging protocols and coils. We found, however, no significant differences in mean values or reproducibility metrics when these two platforms were compared. This implies that multi-center studies may not have to be restricted to a single imaging platform.

To our best knowledge, this study is the first to study the implementation of DCE-CMR in a multi-center setting. We found that significant protocol violation in any of the paired scans could affect as high as 20% of cases, such as using incorrect time interval, insufficient time frames, improper positioning of imaging slabs, and failure to inject contrast agent. Other than that, around 12% subjects were excluded because of poor imaging quality. In this study, because sites were limited to no more than 6 subjects, inexperience was likely a factor in non-compliance. Moreover, the high rate of poor image quality indicates that local operators have difficulties to determine whether the scanned images have adequate quality for quantitative analysis, because DCE-CMR needs customized post-processing and modeling. For future DCE-CMR studies, the following improvements are needed to reduce the dropout rate: 1) optimization of the experience of MR technologists performing the scans, including more intensive hands-on training, mentoring on subject instruction and provision of timely, ongoing feedback for quality control; 2) technological advancements including development of more rapid DCE-CMR protocols, implementation of motion correction reconstruction methods, and improvement in imaging coil design for deep carotid arteries; and 3) strict quality control measures [23], including timely assessment of image quality and imaging protocol adherence at the central review site after post-processing, recall of subjects with poor image quality or protocol violation for repeat scan.

The source of the observed measurement variances may relate to several factors that could also be addressed for further improvement. First, patient positioning differences and subsequent image misregistration between the two scans (Figure 1), even among different time points in DCE series, may be an important factor, because the imaging slice thickness (2 mm) is much larger than the in-plane resolution (0.625 mm). Second, the relatively low temporal resolution (18 s) can introduce variance into the arterial input function extraction. However, the specified spatial and temporal resolution used in this study is a tradeoff with the SNR required to obtain adequate image quality for DCE-CMR analysis. Patient movement during DCE scan can cause blurring and artifacts in images that affect the kinetic analysis. Coil placement differences also can introduce variance. Finally, there may be some natural variability between repeated scans due to perfusion regulation.

One technical limitation of this study is that current analysis of bright-blood DCE images excluded a 1 mm layer near the lumen boundary for measuring mean *K*^*trans*^ and *v*_*p*_, precluding the evaluation of thinner vessel walls. However, this is a tradeoff to minimize the signal contamination in the vessel wall from the high-intensity lumen, which is a major difficulty in bright-blood DCE imaging. Thus, studies using bright-blood DCE techniques are more applicable to larger, more advanced lesions other than small early lesions. Recently proposed black-blood DCE imaging technique may be a solution for thin vessel walls [11],[12],[24]. This study was prospectively designed to estimate scan-rescan measurement errors that have potential utility for future serial imaging studies. Image analysis followed the conventional way in clinical trials where two readers analyze each case sequentially to reach consensus opinion before measurement is locked to avoid human errors and biases. Thus, intra- and inter-reader reproducibility was not assessed. Compared to inter-scan variability, reader variability may be a less problem given that most post-processing steps of DCE-CMR analysis are fully automated. Nonetheless, these metrics need to be studied in future investigations before the technique is used in the clinical setting.

## Conclusions

This study is the first to establish scan-rescan reproducibility and measurement variance of DCE-CMR of carotid atherosclerosis in a multi-center, multi-platform setting. We found both *K*^*trans*^ and *v*_*p*_ derived from DCE-CMR, have moderate reproducibility, the latter with lesions that have a minimum analyzed area greater than 25 mm^2^. Based on these findings, changes in *K*^*trans*^ would be detectable in a prospective study with relatively modest sample size, indicating that it is feasible to run multi-center trials using vessel wall DCE-CMR. However, the rate of uninterruptable images in this study highlights the need for intensive operator training, optimized imaging protocols timely, and ongoing quality control, in future multi-center studies.

## Abbreviations

AIM-HIGH: Atherothrombosis Intervention in Metabolic syndrome with low HDL/high triglycerides: Impact on Global Health outcomes trial

DCE: Dynamic Contrast-Enhanced

CMR: Cardiovascular Magnetic Resonance

ICC: Intra-Class Correlation coefficient

CV: Coefficient of Variation

SD: Standard Deviation

*K*^*trans*^: Transfer constant

*v*_*p*_: Plasma volume

SNR: Signal-to-Noise Ratio

## Competing interests

WSK is a former employee of VPDiagnostics, Inc.

## Authors’ contributions

HC carried out the design of the study, data analysis, data interpretation, and drafted the manuscript. JS participated in study design, data analysis and interpretation, and helped to draft the manuscript; WSK conceived of the study, participated in its design and data interpretation, and helped to draft the manuscript; NB participated in the data interpretation and manuscript revision; MBN performed the statistical analysis, participated the data interpretation and manuscript revision; DSH helped to carry out the statistical analysis, participated in the data interpretation and manuscript revision; DI helped in data acquisition, participated in the data interpretation and manuscript revision; YX participated in the data analysis and manuscript revision; KY participated in the data analysis and manuscript revision; SP participated in the data interpretation and manuscript revision; CY conceived of the study, participated in the data interpretation and manuscript revision; KDO conceived of the study, participated in the study design, data interpretation and manuscript revision; XQZ conceived of the study, participated in the study design, data interpretation, and manuscript revision; All authors read and approved the final manuscript and the submission.
